# Integrating artificial intelligence into medical education: a roadmap informed by a survey of faculty and students

**DOI:** 10.1080/10872981.2025.2531177

**Published:** 2025-07-14

**Authors:** Maria A. Blanco, Sara W. Nelson, Saradha Ramesh, Carly E. Callahan, Kayley A. Josephs, Berri Jacque, Laura E. Baecher-Lind

**Affiliations:** Tufts University School of Medicine, Boston, Massachusetts, USA

**Keywords:** Artificial intelligence, curriculum integration, medical school training, faculty development, teaching and learning

## Abstract

We surveyed faculty and students at a large urban medical school to assess their awareness, usage patterns, and perceived barriers to AI adoption, aiming to identify opportunities for meaningful integration of AI into medical education. We developed a custom survey and distributed it to all medical students (Years 1–4) and a selected group of faculty involved in the MD curriculum. We used descriptive statistics to analyze quantitative data and conducted content analysis on open-ended responses. A total of 128 faculty and 138 students completed the survey. Most participants self-identified as novice AI users and reported limited awareness and infrequent use of AI tools for professional or academic tasks. They cited lack of knowledge, limited time, and unclear benefits as key barriers. Both groups called for training, ethical guidance, and institutional support to facilitate AI integration into medical education. Faculty and students expressed similar needs for targeted AI education, though they emphasized different aspects. In response, our school has conducted a faculty training session and has accelerated identifying opportunities to integrate AI into the curriculum.

## Introduction

Artificial intelligence (AI) is revolutionizing many aspects of modern life, including clinical medicine and medical education. The rapid infiltration of AI throughout healthcare – from diagnosis to documentation – has been accompanied by a surge in literature as clinicians and educators adapt to this evolving environment [1]. Medical educators must adequately prepare students to use AI tools safely and effectively for learning and patient care.

As AI becomes embedded in clinical practice, medical educators face the challenge of incorporating AI literacy, data science, and ethics into the curriculum [2]. Generative AI holds transformative potential across the spectrum of medical education, including admissions, personalized learning, assessment, and research, highlighting the urgent need to enhance AI literacy among medical educators [3]. AI can support medical student learning through personalized feedback, adaptive simulations, and interactive learning environments [4]. Benefits of using AI for these tasks include the immediacy of feedback, enhanced efficiency, cost reduction, and safe environments for clinical skill development. However, challenges such as developing the technology, data privacy concerns, lack of structured curricula, and limited integration into current education systems may prevent more widespread adoption of AI-supported medical education [4].

Medical students worldwide are highly motivated to learn about AI and generally view it as a powerful ally in both education and future clinical practice [5–8]. Despite this enthusiasm, most medical students report minimal or no formal training in AI. Among Canadian medical students, only 15% had received any AI-related instruction by 2022 [9] and similar gaps were more recently observed in India [6,10] and in Palestine [8]. Despite this lack of exposure, students reported wanting structured AI training that is both clinically relevant and ethically informed [6,8–10], including an understanding of AI professionalism and fairness, and how to maintain humanism in this evolving landscape [6,7,11].

Although most studies have focused on student perspectives, several recent studies have included faculty perspectives [7,10,12]. Faculty across multiple regions recognize the importance of AI in medical education and generally support its inclusion in the curriculum. However, they reported being underprepared due to limited familiarity, institutional constraints, and ethical concerns. In Rani et al. [10], 91% of faculty surveyed agreed that AI could enhance medical education, but only 12% reported being ‘very familiar’ with AI technologies. Similarly, Salih [12] found that faculty were interested in using AI in teaching but expressed concerns about their own lack of training and confidence in AI topics. Faculty in this study cited institutional limitations, such as inadequate digital infrastructure and lack of administrative support, as key barriers to AI implementation. They emphasized the need for professional development and institutional investment before AI could be effectively embedded in the curriculum.

Rani et al. [10] highlighted that faculty requested workshops focused on AI basics and pedagogical strategies. In both Salih [12] and Rani et al. [10], faculty expressed uncertainty about how AI might reshape their roles, not just as content experts but also as facilitators of critical engagement with AI outputs. Some feared that AI tools could undermine traditional instructional authority if students relied more on technology than human expertise. These findings highlight a need for structured faculty development initiatives that not only teach AI literacy but also empower educators to guide students in the ethical and effective use of these technologies.

At our institution, the realization that students were independently exploring AI without faculty guidance spurred an institutional effort to assess current use and guide curricular development. We conducted a survey study with faculty and students to explore awareness, usage patterns, and perceived barriers to AI adoption. Our goal was to identify opportunities to support the effective and meaningful integration of AI into medical education. In this paper, we describe our process for initiating the integration of AI into the medical school curriculum informed by findings from our survey of faculty and students.

## Methods

### Context & participants

Our medical school curriculum consists of a 1.5-year pre-clerkship phase followed by a 2.5-year clerkship phase [13]. The pre-clerkship curriculum combines interactive, lecture-based instruction with a variety of active learning modalities, including small-group discussions, team-based learning, and flipped classroom sessions. The clerkship curriculum is delivered across multiple affiliated clinical institutions and includes a one-week interclerkship program as well as simulation-based educational activities conducted at the medical school. In academic year 2023–24, we added a question about AI usage to all student course evaluations to explore how students were using AI for coursework. Data from these evaluations indicated that many students were indeed using AI for learning, even though AI was not a formal part of the curriculum. Following discussions with medical school faculty, as well as a Curriculum Committee retreat on institutional learning objectives, an AI-focused learning objective was adopted by the Curriculum Committee in October 2024. We then conducted a broad survey study with faculty and students to gauge current state of AI use and related needs.

We invited all enrolled medical students from years 1 to 4 (approximately 800 students) to participate in the survey study through class email lists. Additionally, we invited a selected sample of approximately 570 faculty members to participate based on their involvement in the MD curriculum, largely focusing on course and clerkship teaching faculty and faculty coaches. Our basic science faculty also teach students from other health professions, and our clinical faculty are similarly involved in resident education. As such, it is not feasible to draw a strict distinction between faculty roles based solely on learner type. However, for the purposes of this study, we ensured that the survey was distributed specifically to faculty involved in teaching medical students. The survey clearly stated that it focused on faculty experiences with medical students and the medical school curriculum, with the goal of informing AI integration into medical education. This focus was also outlined in the IRB documentation. Recruitment was conducted using email lists associated with various courses, clinical rotations, and the centralized faculty development program. Faculty and student participation was voluntary, anonymous, and no identifiers were collected. The study was determined exempt by the Tufts Health Sciences Institutional Review Board (IRB ID: STUDY00005713).

### Data collection

The research team developed the survey based on a review of the literature conducted by CC, KJ, and the study’s objectives. Our team included faculty, students, and administrators, who collaboratively reviewed and refined the survey items until consensus was reached. The team includes a senior educational researcher with expertise in survey design (MB). Additionally, other team members have received training in and possess relevant expertise in survey methodology (SR, LBL and SN). To ensure clarity and relevance, we pilot-tested the final draft with a select group of twelve resident physicians involved in medical education as a proxy for both the students and faculty perspectives The survey covered topics such as awareness and usage of AI tools, frequency and purpose of use, perceived barriers, and desired support for AI integration. The survey included a mix of question formats, including 5-point Likert scale items, check-all-that-apply questions, and open-ended comment boxes. Administered via Qualtrics from February to March 2025, the survey included up to three reminder emails to encourage participation (Appended Survey Instruments).

### Data analysis

We conducted a comprehensive analysis of the survey data, employing both quantitative and qualitative methods. Descriptive statistics were calculated for the closed-ended survey questions to summarize the data. For the open-ended responses, we performed a content analysis. As primary analysts, MB utilized inductive open coding [14] to identify categorical themes, while SR utilized ChatGPT 4.0 to generate summaries of de-identified comments. The ChatGPT-assisted analysis was validated through multiple prompting iterations, which consistently produced similar key findings. Additionally, all authors independently reviewed the raw comments to identify themes, which were then discussed collectively to reach consensus. Students’ responses were also analyzed by class year to uncover potential similarities and differences among the classes. By triangulating these diverse analytical approaches, we were able to identify common threads and ensure the trustworthiness of our interpretations.

## Results

### AI capabilities, ethical awareness and use

Table 1 displays a summary of the respondents. A total of 128 faculty members responded to the survey (approximately 22% response rate), indicating that they teach a variety of learners, including medical students, residents/fellows, PA students, and graduate students. Regarding medical students’ participation, 138 students responded (approximately 17% response rate), representing a homogenous distribution among classes, with second-year students at the lower end (21%) and the fourth-year students at the higher end (32%). Not all participants responded to every question, resulting in slight variations in total responses by question.

**Table 1.** Summary of survey respondents.

Faculty (*N* = 128)		
	**Learners Taught by Faculty**	
	Medical Students	94%
	Medical Residents and Fellows	56%
	Faculty Members	34%
	Physician Assistant Students	25%
	Graduate Students	16%
	Master of Biomedical Sciences Students	12%
	Doctor of Physical Therapy Students	2%
	Other	6%
	Academic Department*	
	Anatomical and Clinical Pathology	1%
	Anesthesiology and Perioperative Medicine	1%
	Dermatology	1%
	Developmental, Molecular, and Chemical Biology	2%
	Emergency Medicine	6%
	Immunology	2%
	Medical Education	7%
	Medicine	25%
	Molecular Biology and Microbiology	2%
	Neurology	4%
	Obstetrics and Gynecology	5%
	Orthopedic Surgery	1%
	Pediatrics	2%
	Psychiatry	7%
	Public Health and Community Medicine	5%
	Radiation Oncology	1%
	Rehabilitation Services	2%
	Surgery	5%
	Other	9%
**Medical Students** **(*N* = 138)**		
	**Year in Medical School**	
	Year 1 (Class 2028)	24%
	Year 2 (Class 2027)	21%
	Year 3 (Class 2026)	23%
	Year 4 (Class 2025)	32%

*Not all survey respondents answered this question (number of respondents for this item = 122).

**Figure 1.**
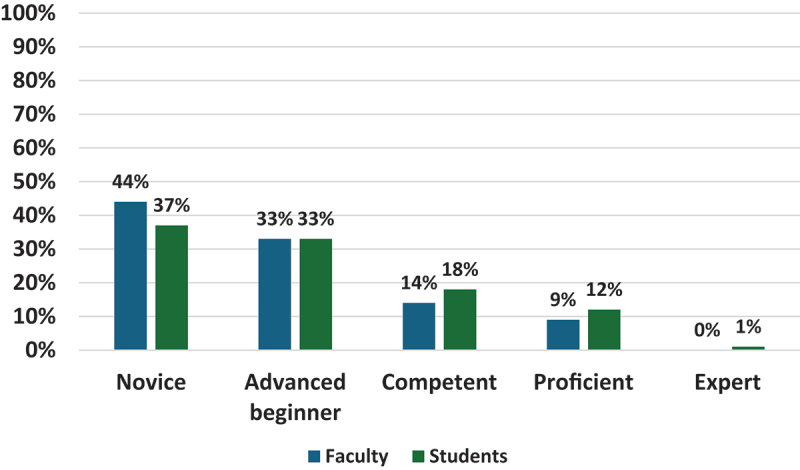
Faculty and students ratings of their understanding of AI capabilities (Faculty = 126; students = 138).

Regarding faculty and students’ ratings of their AI capabilities, in both groups, the majority rated themselves as either novice or advanced beginners, with more students identifying as competent and proficient compared to faculty. One student rated themselves as an expert (See Figure 1).

**Figure 2.**
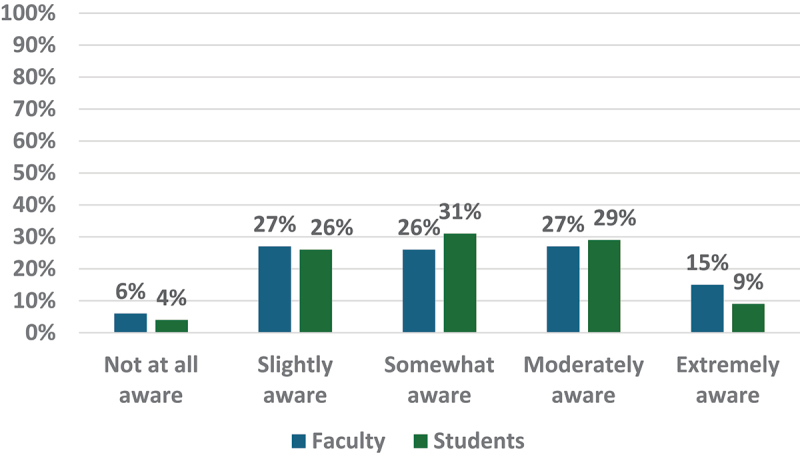
Faculty and students ratings of their awareness of ethical implications using AI (Faculty = 126; students = 137).

Faculty and students’ ratings of their awareness of the ethical implications of using AI ranged from slightly aware to moderately aware, with students’ ratings skewing towards higher awareness within these ranges. Faculty ratings were higher at the extremes of the scale, indicating slightly aware and extremely aware (See Figure 2).

**Figure 3.**
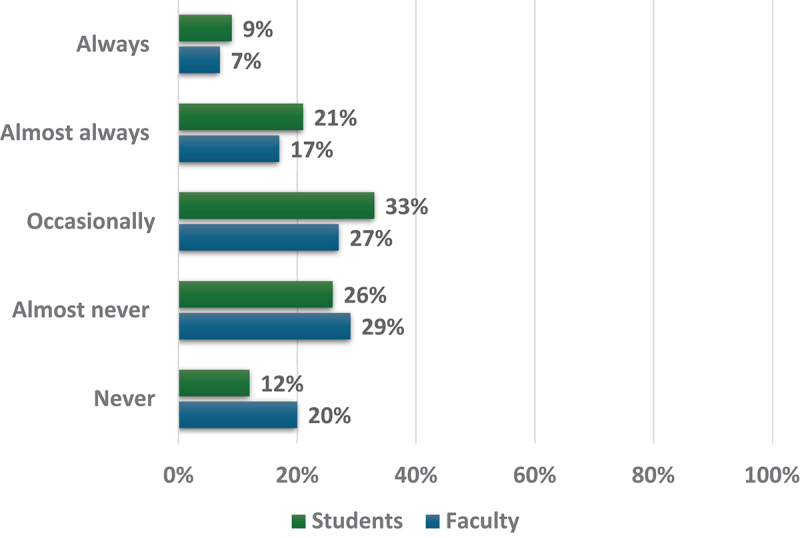
Faculty and students ratings of the frequency of their use of AI tools for professional tasks and medical school related work respectively (Faculty = 126; students = 138).

The majority of faculty (56%) reported that they almost never to occasionally used AI tools for professional tasks. This was followed by 20% of faculty reporting they never used these tools and 7% reporting they always used them. Similarly, 59% of students indicated they almost never to occasionally used AI tools for medical school-related work, followed by 21% who almost always used them and 12% who never used them (see Figure 3). The most frequently used tools among both groups were ChatGPT, UpToDate, OpenEvidence, and DynaMed.

Among faculty that use AI, they reported using AI tools for clinical practice (38%), research literature reviews (28%), curriculum development and content creation (24%), and clinical/classroom teaching activities(17%). Students reported using AI tools for seeking further explanations through interactive learning (52%), enhancing study performance (40%), summarizing with natural language processing (NLP) (34%), practicing questions and exams (29%), and assisting with research (28%).

**Figure 4.**
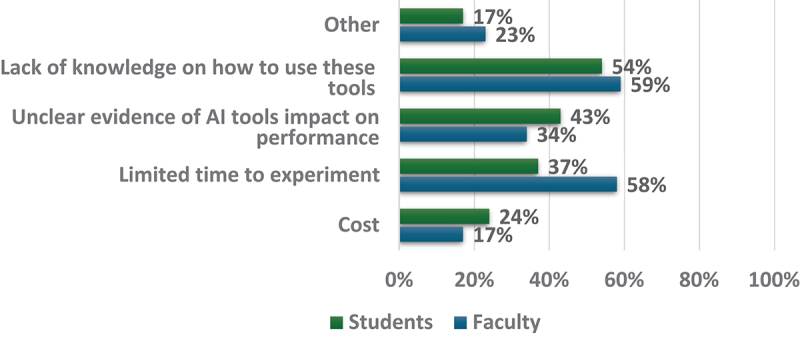
Faculty and students reported barriers for using AI tools (Faculty = 117; students = 123).

As Figure 4 illustrates, when asked about barriers to using AI tools, faculty indicated a lack of knowledge on how to use these tools (59%) and limited time to experiment (58%). Some indicated unclear evidence of AI impact on performance (34%). Students similarly indicated a lack of knowledge on how to use AI tools (54%) and unclear evidence of AI impact on performance (43%), with some also citing limited time to experiment (37%) and cost (24%).

Both faculty and students suggested that the school could better support their use of AI by providing training (67% and 66%, respectively), including hands-on workshops. Faculty highlighted the need for access to free and secure platforms (72%), while students emphasized training sessions on the use of AI for diagnostics (65%), discussions forums on the ethics of AI (48%) and AI platforms for interacting with virtual patients (42%).

### Faculty insights and concerns

The analysis of faculty comments identified ethical concerns, the need for training, and the importance of responsible AI use. Faculty worried that AI might reduce human connection and meaning in work, and benefit tech companies without adequate regulation. Privacy and security concerns specific to healthcare were also raised. Faculty emphasized the need for hands-on AI learning with access to paid tools, training on AI benefits, limitations, and ethics, and fostering responsible AI use by verifying AI-generated content and citations. They also highlighted the importance of developing systems to assess and monitor AI use in education and applying AI cautiously in clinical decision-making. As one faculty notable quote suggested: *‘I’d rather be at the forefront of learning about and using it than be left behind.’*

### Student insights and concerns

Student comments highlighted the changing role of physicians and the need to cultivate AI literacy. Students argued that AI is shifting the role of physicians, making diagnosis less central, and suggested that medical schools should focus on human-centered care and patient counseling. They worry that over-reliance on AI may weaken critical thinking skills and emphasized that AI should support learning without replacing clinical reasoning. Like faculty, students stressed the need for training and resources on effective and responsible AI use, including understanding AI’s strengths, limitations, and ethical concerns. Table 2 presents the specific uses and barriers identified through the analysis of student comments by class year, along with illustrative quotes.

**Table 2.** Specific AI uses and barriers by class year based on student’s comments.

Student Class	AI Uses	AI Barriers	Illustrative Quotes
Year 1 (Pre-Clerkship)	−Basic understanding −Practice material −Thought checks −Process support for reasoning tasks	-Accuracy & trust −Dependence aversion −Environmental impact	*‘It is the BEST individual tutor for my regular classes.’* *‘I do not want to rely on it.’*
Year 2 (Pre-Clerkship)	-Active recall & feedback −Clarification of complex concepts −Literature reviews −Diagnostic support	−Costs -Ethical & moral doubts −Environmental impact −Tools’ limitations	*‘AI would be most useful in offering personalized, rapid iterative feedback.’* *‘I find AI use both morally dubious and the results untrustworthy.’*
Year 3 (Clerkships)	-Clarification & explanation -Brainstorm & creativity −Interactive learning −Practical use for clinical work and study −Planning schedules −Study outlines	−Fear of overuse and dependency −Ethical implications −Environmental impact	*‘AI should be used as Wikipedia – always check the sources.’* *‘Concern that my education will be compromised if I use it and become dependent.’*
Year 4 (Clerkships)	−Content generation −Clinical support -Summarization & review −Research assistance −Assessment preparation -Writing & editing	−Ethical implications −Environmental impact -Accuracy & reliability of information −Professional standards	*‘AI for note writing (humans will never do this again)’* *‘Unclear if it violates professional standards’*

## Discussion

We conducted a survey study to assess the current landscape of AI adoption among faculty and students at our large, urban medical school and to identify opportunities to integrate AI into the curriculum and educational activities.

Our main findings revealed that both faculty and students required targeted education on AI integration in health sciences, highlighting parallel opinions with differences in emphasis among participants. Student and faculty responses showed similar distribution patterns in frequency of use, application of AI tools and ethical awareness. In addition, both groups exhibited a spectrum of views, from strong endorsement to skepticism, highlighting internal diversity.

Consistent with previous studies [5–12], our survey revealed both enthusiasm and ethical reservations regarding AI, as well as widespread educational gaps from the perspectives of both faculty and students. Students highlighted the evolving role of physicians in an AI-integrated future, emphasizing the importance of preserving humanistic care, acquiring diagnostic competencies in AI, and considering the environmental implications of these technologies. Faculty, in turn, underscored their need for training in AI literacy and the implementation of monitored systems to ensure ethical and appropriate use. The trend that students rated themselves as having greater knowledge of AI compared to faculty might illustrate the fact that students, as members of a younger generation, often report greater confidence in their understanding of technology compared to faculty, who may belong to an older generation and have had less exposure to emerging digital tools. This generational difference in technological familiarity may help explain the observed variation in self-reported understanding. Our findings also indicated that students tend to begin using AI as a study aid, gradually incorporating it into clinical and research activities as they progress through medical school. This suggests that AI literacy should be introduced in a developmentally aligned manner. Furthermore, the role of faculty will need to shift from that of content deliverers to facilitators of learning – a role that aligns with the foundational definition of teaching as creating conditions where others can learn effectively with AI serving as a collaborative tool in the educational process. In this context, it may be helpful to clarify the capabilities and functionalities of the AI tools being used, in order to more accurately reflect the true nature of these technologies. Emphasizing how AI can augment human abilities – rather than replace or diminish them – can foster a more balanced and constructive understanding of its role in medical education and practice.

Lastly, it is important to highlight the student’s request for creating forums to discuss the ethical dimensions of AI. As artificial intelligence becomes increasingly integrated into healthcare systems and the practice of medicine, it is reassuring to see that students are not only aware of its potential but also mindful of its ethical implications. Their interest in engaging with these complex issues – particularly how AI might impact patient care, privacy, and decision-making – reflects a thoughtful and responsible approach to the future of medical practice.

We acknowledge the limitation of our survey study, which took place at a single institution among a self-selected groups of students and faculty. As with any survey, there is a potential for response bias, as individuals with particularly strong opinions about AI may have been more likely to participate. Despite this, we used these findings to initiate the integration of AI literacy into our curriculum and hope other schools can use our survey template for their internal needs assessment, as we found no ready-to-go template in the existing literature.

We presented the survey study findings to kick off our first training session with medical school faculty, including course and clerkship directors. We also identified curricular venues to integrate AI formally and seamlessly, such as the Problem-Based Learning course, the Introduction to Clinical Reasoning Course, the Competency-Based Apprenticeship in Primary Care course, and Core Clerkships. Additionally, we continue to analyze a question added to every course and clerkship student evaluation regarding AI use to support learning in the course or clinical rotation. We will continue to offer structured opportunities for faculty hands-on workshops, along with homemade and external online resources for faculty to review at their convenience. In addition, our Student AI Club has been actively organizing discussion forums, including one specifically focused on the ethical implications of AI in medical practice. The medical school has established policies and guidelines for both students and faculty involved in the medical school curriculum regarding AI use, initially drafted in July 2024. These guidelines have since been reviewed and updated for the upcoming academic year to reflect recent technological advancements and insights gained from this survey

Our process and survey experience can inform other schools’ efforts to integrate AI into the medical curriculum, enhance student performance, and improve faculty educational practices. By addressing identified needs and barriers, schools can leverage AI to harness the power of AI tools to empower teaching, learning, and assessment experiences while promoting personalization, co-creation and critical thinking.

## Supplementary Material

Integrating AI in Med Ed Manuscript_Appendix A_Survey Instrument.docx
