# Supplementary material for: Integrating artificial intelligence into medical education: a roadmap informed by a survey of faculty and students
Source: Med Educ Online. 2025 Jul 14;30(1):2531177. doi: 10.1080/10872981.2025.2531177 (PMC12265092; doi:10.1080/10872981.2025.2531177)
Supplement: Integrating AI in Med Ed Manuscript_Appendix A_Survey Instrument.docx [file ZMEO_A_2531177_SM7483.docx]

**Integrating Artificial Intelligence into Medical Education: A Roadmap Informed by a Survey of Faculty and Students**

Blanco MA, Nelson SW, Ramesh S, Callahan CE, Josephs KA, Jacque B and Baecher-Lind LE

**Survey Instrument**

**Purpose:** The purpose of this survey study is to examine the current AI adoption landscape among TUSM faculty and students by assessing awareness, usage and barriers to identify opportunities to support AI integration into TUSM’s curriculum.

**Student Version**

**AI Awareness and Usage**

1. How would you rate your understanding of AI capabilities (e.g. machine learning, natural language processing, computer vision)?

Novice

Advanced beginner

Competent

Proficient

Expert

1. How aware are you of the ethical implications of using AI, such as fairness, transparency, accountability, and the risk of over-reliance on AI?

Not at all aware

Slightly aware

Somewhat aware

Moderately aware

Extremely aware

1. **How frequently do you use AI tools for medical school-related work?**

Never (e.g., you have not used these tools for this purpose at all).

Almost never (e.g., you have used them once every few months over the past year).

Occasionally (e.g., you have used them once every few weeks over the past year).

Almost always (e.g., you have used them multiple times a week).

Always (e.g., you have been using them daily or every time the situation arises).

1. For which of the following purposes do you use AI tools? (Please check all that apply)

Enhance study performance

Customize study plans

Interactive learning to seek further explanations

Natural Language Processing for summaries

Enhanced notetaking

Practice questions and exams

Diagnostic support

Simulated patient cases

Clinical decision-making practice

Efficient clinical documentation

Imaging analysis

Medical knowledge updates

Research assistance

Manuscript/report writing

Networking and career planning

Presentation preparation

Personal branding

Time management

Wellness apps

Financial planning

Health monitoring

Other

Not using any AI tool currently

1. Which of the following AI tools do you use? (Please check all that apply)

AI and Clinical Tools

ChatGPT

Dragon Medical One

Azure AI vision

OpenEvidence

Evidence-based Clinical Decision Tools

UpTodate

Dynamed

VisualDx

Others

NA

1. **In your opinion, which tasks could AI tools be most helpful for in supporting your educational activities in medical school?**[Box for open comments]

**AI Barriers**

1. What barriers prevent you from adopting AI tools? (Please check all that apply)

Cost

Limited time to experiment

Unclear evidence of AI tools impact on performance

Lack of knowledge on how to use these tools

Other

1. **How can TUSM better support your use of AI tools?** (Please select all that apply)

- Classes, courses, or seminars on AI's capabilities, uses, and limitations in medicine
- Sessions on using AI-driven tools for diagnostics
- Hands-on workshops
- AI tutors
- Discussion forums on the ethics of AI
- AI-driven simulation platforms for interacting with virtual patients
- Other:

**Demographic Information**

What is your current TUSM class year?

M25

M26

M27

M28

Other:

Please feel free to share any additional thoughts or insights you may have.

Box for open comments

Thank you for your response!

**Faculty Version**

**AI Awareness and Usage**

1. How would you rate your understanding of AI capabilities (e.g., machine learning, natural language processing, computer vision)?

Novice

Advanced beginner

Competent

Proficient

Expert

1. How aware are you of the ethical implications of using AI, such as fairness, transparency, accountability, and the risk of over-reliance on AI?

Not at all aware

Slightly aware

Somewhat aware

Moderately aware

Extremely aware

1. How frequently do you use AI tools to assist with your professional tasks?

Never (e.g., you have not used these tools at all).

Almost never (e.g., you have used them once every few months over the past year).

Occasionally (e.g., you have used them once every few weeks over the past year).

Almost always (e.g., you have used them multiple times a week).

Always (e.g., you have been using them daily or every time the situation arises).

1. For which of the following purposes do you use AI tools? (Please check all that apply)

Curriculum development and content creation (eg. lecture slides, cases, podcasts, handouts, syllabus, quizzes)

Clinical/classroom teaching activities

Feedback for learners

Grading and evaluation of learners

Screening learner’s applications (eg. to a residency program or rotation)

Plagiarism detection

Research literature reviews

Research design

Data analysis for research

Image analysis

Scholarly writing

Clinical practice (eg. looking up diagnosis and treatments)

Clinical documentation

Time management

Wellness apps

Financial planning

Health monitoring

Travel and scheduling

Other

Not using any AI tool currently

1. Which of the following AI tools do you use? (Please check all that apply)

AI and Clinical Tools

ChatGPT

Dragon Medical One

OpenEvidence

Azure AI vision

PathAI

UpTodate

Dynamed

Visual Dx

Education and Academic tools

Turnitin

Gradescope

Notion AI

Others

NA

1. **In your opinion, which tasks could AI tools be most helpful for in supporting your educational activities?**[Box for open comments]

**AI Barriers**

1. What barriers prevent you from adopting AI tools? (Please check all that apply)

Cost

Limited time to experiment

Unclear evidence of AI tools impact on performance

Lack of knowledge on how to use these tools

Other

1. **How can TUSM better support your use of AI tools?** (Please select all that apply)

- Classes, courses, or seminars on AI's capabilities, uses, and limitations in medical education
- Hands-on workshops
- AI peer coaching
- Discussion forums on the ethics of AI
- Access to secure, free-of-charge platforms for AI tools
- Other:

**Demographic Information**

Which TUSM institution are you affiliated with?

TUSM plus sites drop-down menu

Which department are you in?

Drop down menu with TUSM and clinical departments

What levels of learners do you teach? (check all that apply)

Medical students

PA students

DPT students

Graduate students

MBS students

Medical residents/fellows

Faculty members

Other

Please feel free to share any additional thoughts or insights you may have.

Box for open comments

Thank you for your response!
